# Association between syndecan-1 and renal function in adolescents with excess weight: evidence of subclinical kidney disease and endothelial dysfunction

**DOI:** 10.1590/1414-431X20177174

**Published:** 2018-01-11

**Authors:** Z.M.R.M. Saboia, G.C. Meneses, A.M.C. Martins, E.F. Daher, G.B. Silva

**Affiliations:** 1 Programa de Pós-Graduação em Saúde Coletiva, Centro de Ciências da Saúde, Universidade de Fortaleza, Fortaleza, CE, Brasil; 2 Coordenadoria do Serviço de Saúde, Instituto Federal de Educação, Ciência e Tecnologia do Ceará, Fortaleza, CE, Brasil; 3 Programa de Pós-Graduação em Farmacologia, Faculdade de Medicina, Universidade Federal do Ceará, Fortaleza, CE, Brasil; 4 Programa de Pós-Graduação em Ciências Médicas, Faculdade de Medicina, Universidade Federal do Ceará, Fortaleza, CE, Brasil

**Keywords:** Obesity, Overweight, Biomarkers, Kidney disease, Endothelium, Adolescents

## Abstract

Excess weight (overweight and obesity) is associated with kidney and cardiovascular disease. The aim of this study was to investigate the association between syndecan-1 and renal function among adolescents with excess weight. A total of 56 students from a public school at Fortaleza, CE, Brazil, were investigated. The adolescents were submitted to anthropometric evaluation, including weight, height, blood pressure and body mass index. Blood and urine samples were collected for the determination of serum lipids (total cholesterol, high density lipoprotein cholesterol, low density lipoprotein cholesterol, triglycerides), and the endothelial injury biomarker syndecan-1. Participants' mean age was 16±1 years (range 14-19 years), and 68% were females. Overweight was observed in 4 cases (7.1%) and obesity in 7 (12.5%). Changes in serum lipid levels were more frequent in the overweight group. A positive correlation between syndecan-1 and serum creatinine (r=0.5, P=0.001) and triglycerides (r=0.37, P=0.004), and a negative correlation with glomerular filtration rate (r=-0.33, P=0.02) were found. These findings suggest that adolescents with excess weight present incipient changes at the cellular level that make them more vulnerable to the development of kidney and cardiovascular diseases.

## Introduction

Chronic kidney disease (CKD) is a worldwide public health problem, with increasing incidence and prevalence. The main causes are hypertension and diabetes, but other comorbidities, such as obesity, have been recently indicated as important contributing factors for CKD development (1-3).

The number of people with obesity is also increasing, including in children and adolescents. In a recent large cross-sectional study in Brazil with adolescents, obesity prevalence was 8.4% and it was associated with higher hypertension prevalence (4). We can then consider adolescents with overweight and obesity at increased risk for CKD. Therefore, early diagnosis and screening of CKD is of huge importance to prevent and delay the progression to end-stage kidney disease and decrease morbidity and mortality (2).

The discovery of biomarkers for early identification of cardiovascular and kidney diseases allow diagnosis at initial stages, which in turn allows the adoption of more efficiently measures to slow disease progression and avoid complications. There are currently several kidney injury biomarkers with potential role for early CKD identification (5,6), but there are scarce data in the literature regarding their study among children and adolescents (7,8). As CKD is associated with increased cardiovascular risk (9) as well as obesity, the aim of this study was to investigate the association between syndecan-1 and renal function among adolescents with excess weight.

## Material and Methods

A cross-sectional study was conducted with adolescents studying at the Instituto Federal de Educação, Ciência e Tecnologia do Ceará (IFCE), Campus Fortaleza, Northeast Brazil. The protocol of the study was reviewed and approved by the Ethics Committee of the Universidade de Fortaleza (No. 1.242.456/2015).

All students aged 14 to 19 years old from IFCE were invited to participate in the study, but only 76 agreed to participate. From the 76, only 56 (18 boys and 38 girls) completed all the study phases. Data collection occurred from October 2015 to August 2016 after the participants and their parents signed an informed consent.

The initial phase consisted of an interview and semi-structured questionnaire application containing socio-demographic data, personal and family medical history, life habits, dietary habits and physical activity. Food frequency consumption was evaluated for dairy products, fruits/vegetables and soft drinks. These three food groups were chosen to estimate the consumption of calcium and vitamins, fundamental in the studied age range, as well as sugar and fat consumption, which when ingested in excess is one of the main eating problems of adolescents. A physical examination was then performed by a physician (the first author of this study), including anthropometric measures (weight, height, waist circumference and hip circumference). Overweight was considered for those in between the 85 and 95 percentiles, and obesity for those >95 percentile, according to the most recent recommendations of the Brazilian Society for the Study of Obesity (10). Blood pressure measurements were done in two different moments according to the recommendations of Brazilian guidelines (11).

The second phase consisted of blood and urine collections, which were done by a laboratory technician at a previous scheduled time at IFCE and then sent for biochemical analysis. The following analyses were done: fasting glucose, creatinine, urea, sodium, potassium, chloride, triglycerides, cholesterol, and syndecan-1 (endothelial lesion biomarker). Glomerular filtration rate (GFR) was estimated through the Schwartz formula (12).

Statistical analysis was done with the SPSS program version 20.0 (IBM, USA). Comparison of parameters of the two groups (eutrophic *versus* excess weight) was done with Student's *t*-test and Fischer's exact test. Analysis of associations between excess weight and categorized risk factors was done with Fischer's exact test and Pearson's chi-square test. A logistic regression model was used for quantitative variables. Adjusted odds ratios (ORs) and 95% confidence intervals (CI) were calculated. A multivariate logistic regression was performed to investigate the factors associated with excess weight. The factors included in the multivariate model were those that showed a significance level <20% in the univariate analysis (Mann-Whitney test and chi-square test). Significance level was set on 5% (P<0.05).

## Results

A total of 56 students were included, with mean age of 16±1 years (range 14 to 19 years), and 68% were females. Regarding nutritional status, 45 students were eutrophic (80.4%), 4 (7.1%) overweight, and 7 (12.5%) obese. Mean body mass index (BMI) was 23±4.5 and waist circumference 73.6±15.9 cm.

Regarding clinical characteristics between eutrophic *vs* excess weight students, there were significant differences in weight, BMI, waist circumference, hip circumference and blood pressure (Table 1). In relation to blood pressure, 9 students (20%) in the eutrophic group had pre-hypertension, while 4 (36.3%) in the excess weight group had this diagnosis. Hypertension was found in 1 student (2.2%) in the eutrophic group and in 3 (27.3%) in the excess weight group. Blood pressure was the only independent variable associated with excess weight: systolic blood pressure (OR=1.08, 95%CI=1.01-1.16, P=0.02) and diastolic blood pressure (OR=1.16, 95%CI=1.03-1.31, P=0.009). None of the adolescents had previous diagnosis of arterial hypertension, and those who were diagnosed during the study were referred for outpatient follow-up in a specialized service.

**Table 1.** Comparison of clinical characteristics of adolescents with and without excess weight (overweight and obesity) in a public school in Fortaleza, CE, Brazil, October 2015 to August 2016.

	Eutrophic (n=45)	Excess weight (n=11)	P
Age (years)	16±1.2	15.5±1	0.05
Weight (kg)	54.4±8.3	79.1±11.7	<0.001
Height (m)	1.60±0.08	1.63±0.09	0.783
BMI (kg/m^2^)	21.2±2.2	29.8±3.5	<0.001
Waist circumference (cm)	67.9±13.3	90.6±10.7	<0.001
Hip circumference (cm)	89.4±16.3	109.9±6.1	<0.001
Waist/Hip circumference ratio	0.76±0.05	0.83±0.07	0.001
Systolic blood pressure (mmHg)	111±10	121±14	0.01
Diastolic blood pressure (mmHg)	71±7	79±6	0.003
Normal blood pressure	35 (77.8%)	4 (36.4%)	0.01
Pre-hypertension	9 (20%)	4 (36.3%)	0.25
Hypertension	1 (2.2%)	3 (27.3%)	0.02

Data are reported as means±SD or number (percentage). BMI: body mass index. Statistical analysis was done with the chi-squared test and Student's *t*-test.

**Figure 1.**
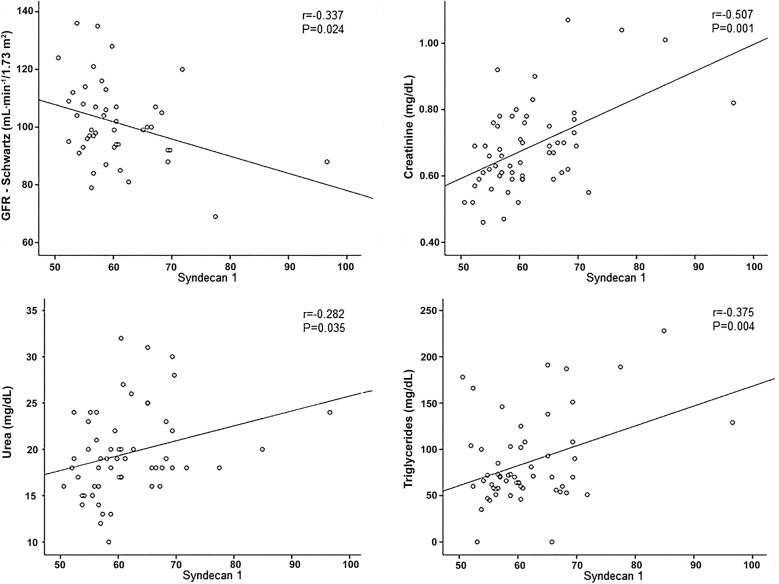
Correlation between syndecam-1 and glomerular filtration rate (GFR), creatinine, urea and triglycerides among adolescents with excess weight (overweight and obesity) in a public school in Fortaleza, CE, Brazil, October 2015 to August 2016.

Laboratory tests had no differences regarding renal function between the eutrophic and excess weight groups (creatinine=0.67±0.11 *vs* 0.70±0.16 mg/dL, P=0.53; GFR=102±12 *vs* 100±20 mL·min^-1^/1.73m^2^), and the levels of HDL cholesterol were lower among excess weight adolescents (45±9.3 *vs* 35±9 mg/L P=0.002). There was no significant difference regarding the other laboratory tests between the two groups. Syndecan-1 levels presented a significant negative correlation with GFR and a positive correlation with creatinine, urea and triglycerides (Figure 1). There was no significant abnormality in urinalysis.

The average family income of the majority of students (59%) was between 1000 and 3000 Brazilian reals per month (middle income class), with overweight students predominantly from the lowest income class (45%). In relation to practice of physical activities, 47 students (83.9%) were active, including the majority of overweight students (72.7%). There was a high frequency of consumption of full-fat dairy products and soft drinks by the majority of students and inadequate consumption of fruits and vegetables in relation to the number of servings/day.

## Discussion

The present study found a high proportion of excess weight in adolescents (19.3%) and a significant correlation between renal function and endothelial damage, evidenced by syndecan-1 levels, indicating a possible subclinical kidney injury and endothelial dysfunction.

Past studies have shown associations between high BMI and risk of cardiovascular and kidney diseases (13). Early identification of adolescents at risk for kidney disease is very important to allow the adoption of preventive measures, including lifestyle modifications and blood pressure control. New biomarkers would be essential to achieve this purpose.

In the present study, hypertension was more frequent in the adolescents with excess weight, which is in accordance with the literature (4). In the multivariate analysis, both systolic and diastolic blood pressure were independent factors associated with excess weight. Another important abnormality frequently found among excess weight adolescents was lower HDL cholesterol levels.

Syndecan-1 was increased in adolescents with excess weight in our study, and had a significant correlation with traditional renal function markers (GFR, creatinine and urea) and lipids (triglycerides), which indicates subclinical kidney and vascular injury and a higher cardiovascular risk, as a syndecan-1 increase is a result of glycocalyx damage (14). Endothelial glycocalyx markedly changes its properties under inflammatory conditions, contributing to the reduction of renal function. Discarded components of glycocalyx can therefore be considered a very early sign of endothelial activation, which occurs in inflammatory states. In agreement with this proposal, syndecan-1 and heparan sulfates have been shown released from the endothelium following stimulation with thrombin or endotoxin (15).

These results point to the occurrence of an insidious endothelial dysfunction in individuals with excess weight, even in those with normal levels of traditional markers. Early identification of kidney and endothelial dysfunctions is important because they are associated with a high cardiovascular risk, allowing adoption of more effective preventive measures and a more careful follow-up of these individuals. The identification of adolescents at high cardiovascular risk has importance because individuals in this age group are more susceptible to preventive measures and lifestyle modifications that could impact not only in retarding kidney disease progression but also in decreasing the incidence of chronic diseases, including hypertension and other cardiovascular diseases.

The main limitation of this study is the small sample size. We had difficulty in recruiting asymptomatic adolescents to undergo medical history assessment and physical examination and to collect biological material (blood and urine). They were all adolescents with good general health but were not willing to help with scientific research. However, we have found interesting results, which showed subclinical abnormalities indicating an increased cardiovascular risk of adolescents with excess weight. The novel biomarkers investigated here seem to be useful in the early identification of renal and endothelial damage in adolescents with excess weight. These biomarkers might be used for the screening of cardiovascular disease in this group.

The results of this research only apply to this population, since the number of participants was small. However, considering the clinical importance of the discovery of novel biomarkers of kidney disease and their relationship with the obesity epidemic, new longitudinal studies in this area with a larger number of participants are mandatory.
